# The Feasibility of Two Handheld Spectrometers for Meat Speciation Combined with Chemometric Methods and Its Application for Halal Certification

**DOI:** 10.3390/foods11010071

**Published:** 2021-12-29

**Authors:** Abolfazl Dashti, Judith Müller-Maatsch, Yannick Weesepoel, Hadi Parastar, Farzad Kobarfard, Bahram Daraei, Mohammad Hossein Shojaee AliAbadi, Hassan Yazdanpanah

**Affiliations:** 1 Department of Toxicology and Pharmacology, School of Pharmacy, Shahid Beheshti University of Medical Sciences, Tehran P.O. Box 14155-6153, Iran; abolfazl.dashti@sbmu.ac.ir (A.D.); bdaraei@sbmu.ac.ir (B.D.); 2 Food Safety Research Center, Shahid Beheshti University of Medical Sciences, Tehran P.O. Box 14155-6153, Iran; 3 Wageningen Food Safety Research, Wageningen University and Research, P.O. Box 230, 6700 AE Wageningen, The Netherlands; judith.mueller-maatsch@wur.nl (J.M.-M.); yannick.weesepoel@wur.nl (Y.W.); 4 Department of Chemistry, Sharif University of Technology, Tehran P.O. Box 11155-9516, Iran; h.parastar@sharif.edu; 5 Department of Medicinal Chemistry, School of Pharmacy, Shahid Beheshti University of Medical Sciences, Tehran P.O. Box 14155-6153, Iran; farzadkf@yahoo.com; 6 Faroogh Life Sciences Research Laboratory, Tehran P.O. Box 14578-34491, Iran; farooghlab@gmail.com

**Keywords:** handheld VIS/NIR, halal meat, chemometrics, meat, speciation, authenticity

## Abstract

Handheld visible-near-infrared (Vis-NIR) and near-infrared (NIR) spectroscopy can be cost-effective, rapid, non-destructive and transportable techniques for identifying meat species and may be valuable for enforcement authorities, retail and consumers. In this study, a handheld Vis-NIR (400–1000 nm) and a handheld NIR (900–1700 nm) spectrometer were applied to discriminate halal meat species from pork (halal certification), as well as speciation of intact and ground lamb, beef, chicken and pork (160 meat samples). Several types of class modeling multivariate approaches were applied. The presented one-class classification (OCC) approach, especially with the Vis-NIR sensor (95–100% correct classification rate), was found to be suitable for the application of halal from non-halal meat-species discrimination. In a discriminant approach, using the Vis-NIR data and support vector machine (SVM) classification, the four meat species tested could be classified with accuracies of 93.4% and 94.7% for ground and intact meat, respectively, while with partial least-squares discriminant analysis (PLS-DA), classification accuracies were 87.4% (ground) and 88.6% (intact). Using the NIR sensor, total accuracies of the SVM models were 88.2% and 81.5% for ground and intact meats, respectively, and PLS-DA classification accuracies were 88.3% (ground) and 80% (intact). We conclude that the Vis-NIR sensor was most successful in the halal certification (OCC approaches) and speciation (discriminant approaches) for both intact and ground meat using SVM.

## 1. Introduction

The authenticity of food such as meat and meat products has become a growing demand throughout the world [1]. Counterfeit of common livestock meat products has caused serious social issues among religious-concerned consumers, allergic consumers and also deteriorates the credit of the enterprises. As an example of religious concern, Islamic and Jewish laws forbid the consumption of pork, while many Hindus do not consume any product from cows [2].

The market for halal-certified products is increasing globally, including non-Muslim countries, selling not only raw meat products but processed food in supermarkets [3,4]. When it comes to halal meat, several differences to the commercial meat available in non-Muslim countries, especially in Europe and the United States, are found. Halal meat may only contain meat from ruminant species such as cows, sheep, goats or camel [5]. Pork is not considered halal, whilst poultry meat from some species such as chickens, geese, ducks and turkeys is allowed. In addition to that, halal meat may only be retrieved from a slaughtering process that ensures ritual rules of slaughtering including the complete drainage of blood [5]. The diagnostics of halal and non-halal meat according to the animal species and slaughtering procedure require complex testing and certified paper trails. As a result, Ruslan et al. [6] and McElwee et al. [7] have reported several cases of halal meat fraud. According to the conclusion of both studies, the detection of halal meat authenticity is the main issue in detecting halal meat fraud.

The demand for authenticity and safety has led to a higher need for quality assurance in the meat production system. Different DNA and protein-based methods, such as polymerase chain reaction (PCR), chromatographic and mass spectrometry (MS) techniques were developed for meat authenticity [8,9]. Spectroscopic methods such as handheld visible (VIS) and near-infrared (NIR) spectroscopy, or a combination thereof, have great potential for food authenticity testing as a rapid, low-cost, non-destructive and transportable technique [8,10]. Handheld spectroscopic instruments could screen food authenticity in a simple, rapid way in-situ and therefore bring a great advantage compared to conventional techniques such as PCR, liquid chromatography and MS. They permit very quick intervention possibilities in fraudulent case detection without elaborated sample preparation and long analysis times. Spectroscopy can therefore be used for screening purposes to efficiently select samples to be sent to the laboratory for confirmatory analysis [11,12,13]. Nowadays, developing a low-cost, rapid, non-destructive method with a handheld sensor that can be implemented along the entire food supply chain has become an area of major interest [13,14,15,16]. These studies have focused on the application of handheld spectroscopy sensors for the determination of authenticity of spices [17], honey [18], olive oils [19], and beef adulterated with pork and chicken [20].

Handheld VIS and NIR spectrometers have a relatively small spectral range and low resolution in contrast to benchtop spectrometers most of the time. Moreover, scattering effects and ambient and instrumental noise make robust chemometric methods crucial for the extraction of relevant information from obtained spectra [8]. Authentication studies using handheld spectrometers usually assign samples to one or more categories or classes. To do so, multivariate classification methods are used for supervised data modeling. In this regard, the two frequently used multivariate classification techniques consist of discrimination methods (e.g., partial least squares discriminant analysis, PLS-DA) and class modeling methods (e.g., soft independent modeling of class analogy, SIMCA). The selection of the correct strategy depends on the final goal of the screening method. Usually, when there are at least two different classes, the discriminant approach is suitable, while class modeling is appropriate when a single class is the focus [21,22]. In the discriminant models, new samples are always allocated to the class to which they probably belong, even in the case of objects that are not related to any class studied. These methods can be linear (e.g., PLS-DA) or non-linear (e.g., SVM (radial base function) RBF kernel)) [23]. Class modeling can be used when there is only one class of interest, and for this reason, this method is also called one-class classification (OCC). Class modeling methods determine a closed class space around the samples and their shapes depend on the type of modeling methods and the width is related to a pre-defined confidence level. When a new sample falls inside this enclosed space, it is considered compatible with the class, while samples falling outside are considered extraneous. They provide an answer to the general question: “Is sample X, claimed to belong to class C, really compatible with the class C?” [22,24].

Regarding halal meat certification, there are two main issues: the first one is meat speciation meaning it is made of only Halal species such as ovine or bovine, and equally important is that non-halal species (such as pork) shall not be present; secondly is to establish the Islamic ritual slaughtering method according to the acceptable standards (the Organization of the Islamic Cooperation/Standards and Metrology Institute for the Islamic Countries (OIC/SMIIC) 1: 2019, General Requirements for Halal Food to be eligible as Halal Authenticity Screening Test Methods) and Codex Alimentarius Commission. More details about halal certification can be found in references [5,25]. In the case of halal meat species detection, the target class may be set as pork, which is non-halal meat. That means that all samples that are “in”, do contain pork and therefore are not halal. On the other hand, all samples that are “extraneous” do not contain pork and may be labeled as halal. In this case, we can fulfil the first requirement laid down by the OIC/SMIIC, General Requirements for Halal Food to be eligible as Halal Authenticity Screening Test Methods [5].

This study aims to evaluate and compare the capability of handheld Vis-NIR and handheld NIR sensors combined with chemometric methods for speciation of four different types of meat (lamb, beef, pork and chicken) as well as halal meat species certification (pork vs. other species). In this study, the halal species (sheep, calf and chicken) are slaughtered according to Islamic rules and, therefore, the collected meat samples were halal. We show the application of OCC and discriminant chemometric approaches (linear and non-linear) on data obtained using handheld sensors. Meat samples, especially those in the intact form, have a heterogeneous nature that influences the performance of NIR spectroscopy prediction models [10,26,27]. According to the heterogeneous nature of meat and the existence of meat in both forms in retail, we decided to evaluate the feasibility of handheld sensors for intact and ground forms. In addition, for covering the variety of meat cuts, samples were collected from different parts of the animals. Furthermore, the performance of different preprocessing techniques, cross-validation (internal validation) strategies and data splitting approaches on the ability of discrimination techniques were evaluated.

## 2. Materials and Methods

### 2.1. Sample Collection and Preparation

For NIR analysis, 48 beef (*Bos taurus*) muscle samples (fore and hind shank, chuck, brisket and round), 41 lamb (*Ovis aries*) muscle samples (fore and hind shank) and 40 chicken (*Gallus gallus domesticus*) muscle samples (breast and drumstick) were obtained 24 h after slaughtering from local butchers in different cities of Iran. Additionally, 32 pork (*Sus scrofa domesticus*) muscle samples (shoulder and leg) were collected from Azerbaijan. All meat samples were purchased in intact form and transported chilled to the laboratory.

For Vis-NIR analysis, forty beef muscle samples (fore and hind shank, chuck, brisket and round), thirty-seven lamb muscle samples (fore and hind shank), and forty chicken muscle samples (breast and drumstick) were obtained from local butchers in different cities of Iran. Moreover, 40 pork muscle samples (shoulder and ham) were collected from local butchers around Wageningen, the Netherlands. All samples were analyzed after at least 24 h postmortem. Each sample was obtained from a different animal to ensure a variety of samples representing one type of meat. However, age and sex were not considered as additional variability factors. Fresh meat samples were stored at 0–4 °C until the analyses. This study consisted of two parts: first, classification of intact (non-ground) meat and second, classification of ground meat. Intact samples were prepared by cutting slices and both intact and ground meat were placed in a Petri dish (1 cm deep, 6 cm diameter) and the excess of a sample was adjusted with a filling knife to obtain a smooth surface until scanned.

For the second part, visible skin and fat that could interfere in the analysis were removed with a kitchen knife and then about 100–200 g of meat was homogenized for 30 s with a Moulinex Meat Grinder (1000 W). The blender cup was washed first with hot water and detergent, then with cold water, followed by drying with a towel between samples.

### 2.2. Data Acquisition

#### 2.2.1. NIR

NIR spectra were acquired with a TellSpec Food Sensor (TellSpec Inc., Toronto, ON, Canada) (82.2 mm × 66 mm × 45 mm, weight 136 g), in reflectance mode in the wavelength range of 900–1700 nm with an evenly distributed spectral resolution (3 nm), resulting in 256 variables/measurement. Samples were analysed immediately after they were taken out of the fridge, with a sample temperature of 4–7 °C. Spectral acquisitions were performed on six different points of each intact and/or ground sample (5 at the edges of the sample and 1 at the center). The acquisition of the spectra was performed in the same region for all samples. The handheld NIR data matrix consisted of 966 spectra (161 samples × 6 points) with 252 wavelengths.

#### 2.2.2. Vis-NIR

Vis-NIR spectra were acquired in the range of 400–1000 nm with 600 spectral bands using a LinkSquare handheld Vis-NIR spectrometer from Stratio Inc. (Stratio Inc., San Jose, CA, USA) in reflectance mode. LinkSquare is a silicon (Si)-based Vis-NIR spectrometer (114.0 × 23.9 × 23.9 mm, weight 57 g). The spectrometer has two light sources, one being a white light-emitting diode (LED) and a bulb light source for covering the respective visible and infrared range [28]. For data analysis, two derived spectra from each scan were concatenated together. The spectral range of the spectrometer was from 400 to 1000 nm with a resolution of 1 to 20 nm (approximately 1 nm at 600 nm, 4 nm at 800 and 16 nm at 1000 nm). Samples were analysed immediately after they were taken out of the fridge, with a sample temperature of 4–7 °C. Finally, the handheld Vis-NIR data matrix consisted of 942 spectra (157 samples × 6 replicates) with 1200 points (variables). For both sensors, the spectral acquisition was obtained from a maximum 5 mm distance (according to the sensors instruction) without any contact to the surface of the sample for cross-contamination.

### 2.3. Data Analysis

Chemometric data analysis was performed in MATLAB (Mathworks, MA, USA) and R software. For discriminant analysis and pre-processing of raw data, the PLS-Toolbox version 7.8 (Eigenvector, WA, USA) and Classification Learner toolbox were used.

Training (70%) and test (30%) sets were produced using the Duplex method [8,29] to ensure that all species were represented in the test set. The effect of different data splitting methods such as duplex, Kennard-stone and random algorithms were evaluated for splitting data to the training set and test set. Finally, for having the same diversity in both sets (train and test), the duplex algorithm was used for data splitting.

**Figure 1:**
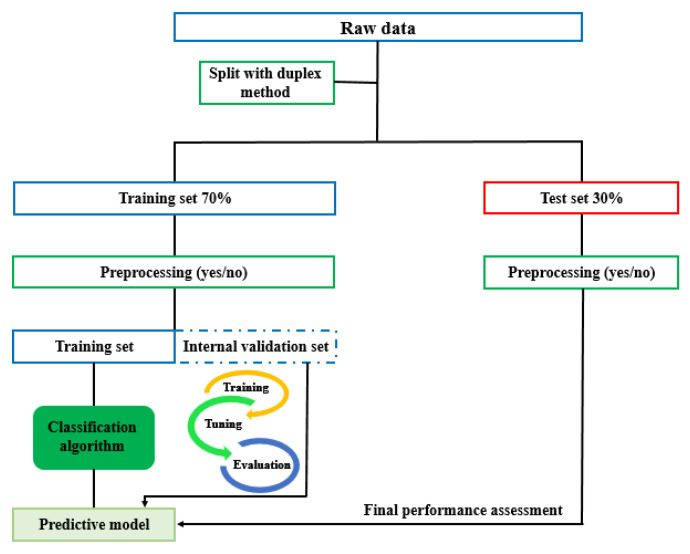
Data analysis pipeline.

To ensure that the test set did not include data from the same sample that the model was trained on, all spectra of one sample were allocated to either the training set or test set. The pre-processing of VIS-NIR raw data was the first step of model development and optimization and should improve the subsequent classification model or data exploratory analysis [30,31,32]. Therefore, before classification, the raw data was pre-processed with different methods such as multiplicative signal correction (weighted normalization and baseline removal and median ratio normalization), standard normal deviate (SNV) correction, 1st derivative, 2nd derivative (Savitzky–Golay and gap segment, 5, 11, 15 and 21-point filter length), mean centering, OSC (orthogonal signal correction) and baseline filtering. In this study, the best pre-processing combinations were selected based on the best separation between samples in a PCA (principal component analysis) scores plot and also the best performance parameters for discrimination/class modeling methods. The predictive classification models were validated using cross-validation (Venetian blinds (10 splits and 1 sample per split) and 5-fold cross-validation). After training, tuning and evaluation of the model, the test set was used for the final performance assessment. The data analysis pipeline of the presented work is shown in Figure 1. As part of this study, the effect of different cross-validation methods including Venetian blinds cross-validation, leave-one-out cross-validation (LOOCV) and random sub-sets cross-validation (RSSCV) were evaluated as an example in ground meat NIR spectra. Finally, Venetian blinds cross-validation was used for internal validation because this method has a lower tending to overestimation of the predictive power of the model and could return a reliable number of latent variables [33,34].

#### 2.3.1. OCC Approach

The approach performed with R 3.6.1 (R Core Team, Vienna, Austria) was described in detail by Weesepoel et al. [19] and Mueller-Maatsch et al. [35] when detecting the authenticity of extra virgin olive oil and skimmed milk powder, respectively. In brief, a systematic screening of data pre-processing steps, in particular, SNV, SNV followed by baseline correction (SNV, detrend, DT), 1st or 2nd derivative (Savitzky-Golay) with an 11-point filter length, discrete wavelet transformation (DWT) after interpolating the spectrum into 128 points (returning the 5th–7th level wavelet coefficients from a Daubechies with filter length 2 or the 3rd-5th level least asymmetric with filter length 8), and spectrum splitting (either full or split into 4 equal quarter spectra modeled separately) were performed. In addition, the following one-class classification algorithms were evaluated: SIMCA, distance to k-Nearest Neighbour (kNN), PCA residual, Mahalanobis distance, One-Class Support Vector Machine (OCSVM) with RBF kernel. Each combination of pre-processing and classification algorithm was evaluated using an 80 times repeated random cross-validation (70% split) on the target class (pork), where sample replicates were kept together in test and training sets. Performance was evaluated by calculating the ‘area under the receiver operating characteristic’ (AUROC) of the target class pork against lamb, beef and chicken, respectively. Three models were selected manually as they jointly covered the highest obtained AUROCs for each of the classes. The median class distance of the 6 replicates was used in the final classification: a simple decision tree, i.e., if two or more out of the 3 models classified a sample as ‘out-of-class’ it was flagged as ‘not pork meat’. Other traits for determining the class distances were not tested.

#### 2.3.2. Discriminant Approach

Discriminant methods needed at least two classes and classified unknown samples in the nearest class [33,36]. In this study, two discriminant methods including PLS-DA and SVM were used. In PLS-DA, the optimal number of PLS factors (latent variables (LVs)) for the models was selected by Venetian blinds cross-validation (number of data split: 10, thickness: 1). Other attempts such as outlier detection using Q-residuals/Hotelling’s T^2^ [33] and variable selection using variable importance in projection (VIP) with the “greater than one” rule [37] were conducted to increase PLS-DA classification performance. For SVM parameters optimization, different methods can be used. All these methods are generally based on a cross-validation classification rate to appraise the performance of the model and minimize the risk of overfitting [38,39,40]. In the current study, RBF, quadratic and cubic kernels as non-linear kernel-function were tested.

The performance of classification models was assessed according to the percentage of samples truly classified during calibration development and, afterward, with external validation. These performance parameters, such as sensitivity, specificity, accuracy and error rate are usually derived from a confusion matrix, to better assess the classification performance. The parameters were computed according to the following expressions combining the number of true positives (TP, correctly identified), true negatives (TN, correctly rejected), false positives (FP, incorrectly identified) and false negatives (FN, incorrectly rejected) obtained in calibration and validation. These parameters are defined according to Equations (1)–(4) [33,41]:

(1) $$\;\mathrm{Sensitivity}=\frac{\mathrm{TP}}{\mathrm{TP}+\mathrm{FN}}$$

(2) $$\;\mathrm{Specificity}=\frac{\mathrm{TN}}{\mathrm{TN}+\mathrm{FP}}$$

(3) $$\mathrm{Accuracy}=\frac{\mathrm{TP}+\;\mathrm{TN}}{\mathrm{TP}+\mathrm{TN}+\mathrm{FP}+\mathrm{FN}}$$

(4) $$\;\mathrm{Error}\;\mathrm{rate}=1-\mathrm{Acc}=\frac{\mathrm{FP}+\mathrm{FN}}{\mathrm{TP}+\mathrm{TN}+\mathrm{FP}+\mathrm{FN}}$$

Furthermore, leave-class-out (LCO) was used as a validation method for the developed model. In LCO, one class was left out from the original data set and the model was built using the remaining data. Then, the left-out class was imported into the model as a test set. Several models were trained (and cross-validated) using 4 − 1 = 3 classes. This method was repeated four times. As there is no ‘correct classification’ in the LCO method, cut-off (0.5) was imposed on the classification probability, before a classification would be accepted [8]. It should be pointed out that LCO validation was performed only for the SVM model for ground meat data because its performance was slightly better than PLS-DA.

## 3. Results and Discussion

### 3.1. Intact and Ground Meat Spectra

**Figure 2:**
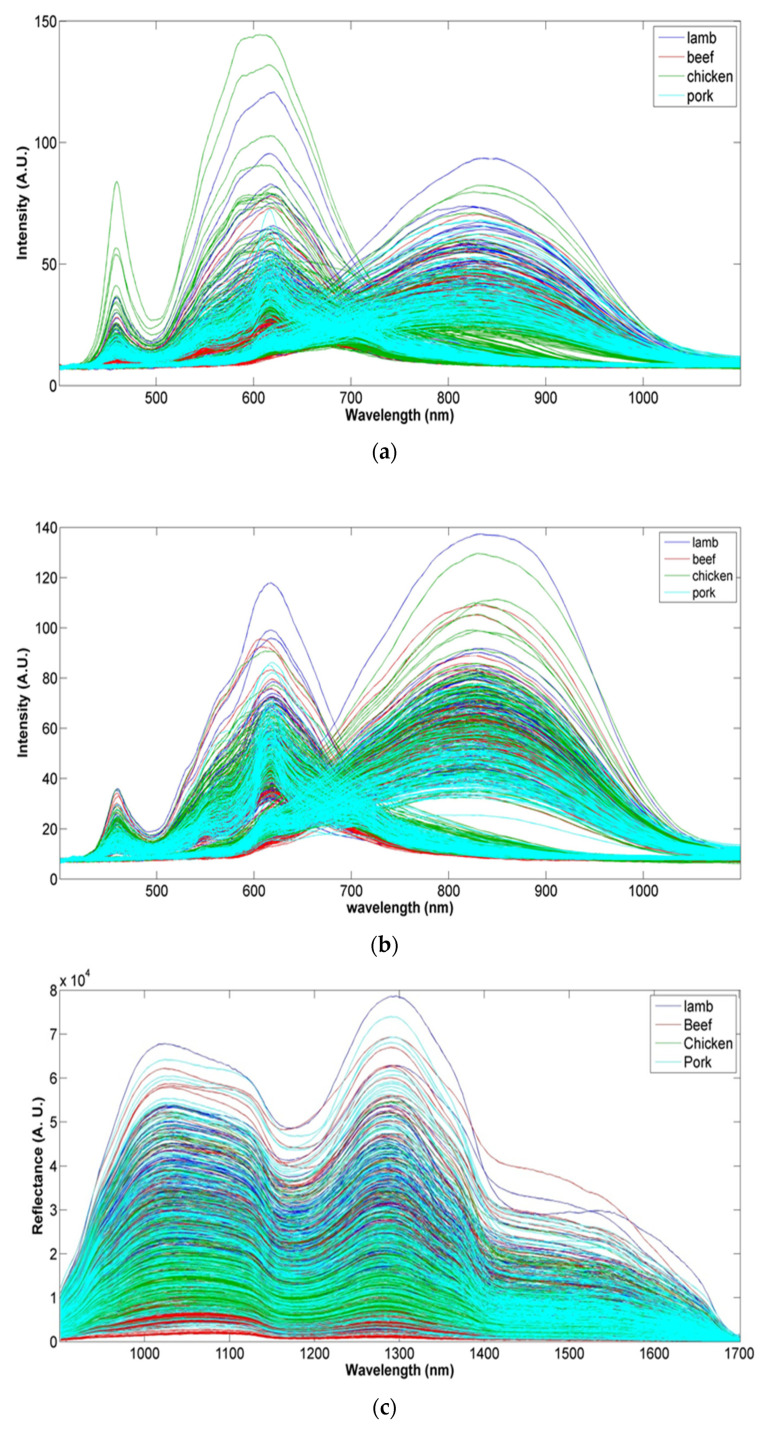

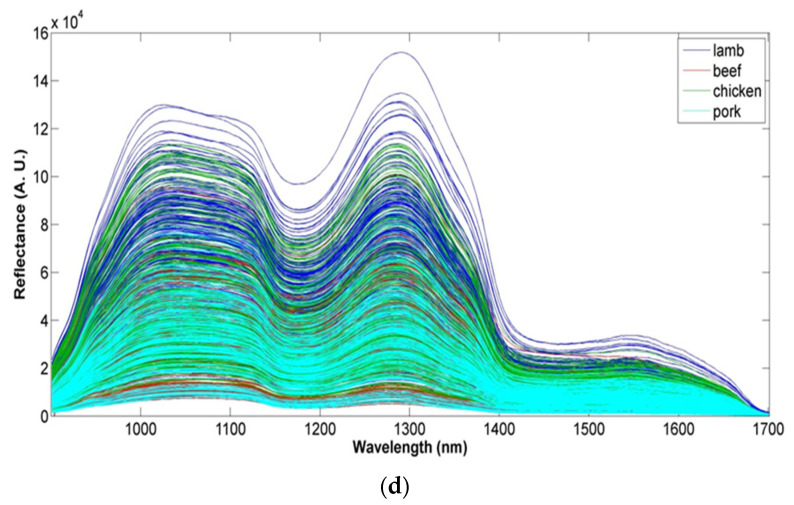
(**a**) Vis-NIR spectra of intact meat with smoothing, (**b**) Vis-NIR spectra of ground meat with smoothing, (**c**) raw NIR spectra of intact meat, (**d**) raw NIR spectra of ground meat. Blue: lamb, red: beef, green: chicken, turquoise: pork.

The total spectra of the intact and ground meat of lamb, beef, chicken and pork collected with Vis-NIR and NIR sensors are shown in Figure 2. Regarding Vis-NIR spectra, visual differences were observed between sample species in the visible (respiratory pigments) and NIR regions. Peaks around 418, 546 and 578 nm may be associated with myoglobin and hemoglobin absorption (Figure 2a,b) [42,43]. Spectra collected with the NIR sensor on the intact and ground meat (Figure 2c,d) were characterized by absorption bands at 1450 nm attributable to the first overtone of OH, and also present at an absorption band at 1200 nm related to the second overtone of C–H aliphatic group stretching [44,45]. In all samples, the spectra in Vis-NIR and NIR were very similar and small differences could be observed visually between averaged spectra of the four considered meat species. Therefore, the application of chemometric methods was necessary for the determination of the feasibility of the sensors for meat speciation purposes.

### 3.2. Exploratory Data Analysis Using PCA

**Figure 3:**
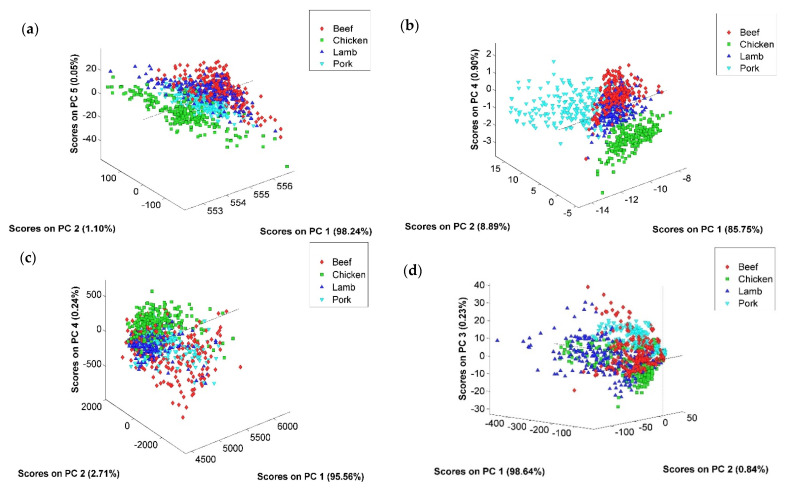
Vis-NIR data (400–1000 nm): (**a**) The PCA score projections of Vis-NIR spectra of intact meat preprocessed with EMSC; (**b**) the PCA score projections of Vis-NIR spectra of ground meat preprocessed with EMSC + 1st derivative. NIR data (900–1700 nm): (**c**) The PCA score projections of NIR spectra of intact meat preprocessed with MSC (mean) + 1st derivative (SavGol); (**d**) the PCA score projections of NIR spectra of ground meat preprocessed with gap segment 2nd derivative.

For data exploration and assessment of the similarities and differences, the Vis-NIR and NIR data were subjected to PCA (Figure 3). For Vis-NIR spectral data, after evaluating the different pre-processing algorithms, the best PCA models (best separation between species) were developed using extended multiplicative signal correction (EMSC) for intact meat and EMSC plus 1st derivative for ground meat (Figure 3a,b). For intact and ground meat, the 1200 spectral variables were reduced into two significant principal components, PC1 and PC2, which explained 99.3% and 94.6% of the cumulative variance in the data, respectively. In the intact and ground meat PCA score plot, PC1 is corresponding to the maximum variance but the best separations (clustering) between species have appeared on the PC1 and PC2 for intact and ground meat, respectively (Figure 3a,b).

For NIR spectral data, it was found that the MSC (mean) combined with 1st derivative (SavGol, 11-point filter length) for intact meat and the gap segment 2nd derivative (gap: 5, segment: 5) for ground meat were the best data pre-processing methods for relative species separation in PCA score plots. The score plot of PC1 (95.6%) vs. PC2 (2.7%) and PC4 (0.2%) for intact meat, and PC1 (98.6%) vs. PC2 (0.8%) and PC3 (0.2%) for ground meat (Figure 3c,d) show that the variation was best explained in the first PC. The inadequate separation between the species in PCA score plots of NIR spectra indicates similarity in their spectral patterns; however, there are numerous aspects (e.g., physical and chemical) that could differ leading to slight spectral differences. The causes for the differences are most likely ascribed to the variation of macronutrient composition [46].

### 3.3. OCC Approach

In this approach, three models (Table 1 and Table 2) were selected manually as they covered jointly the highest obtained AUROCs for each of the classes. Different class limits may be applied to the selected models leading to different scenarios. For example, in Table 3, scenario 1 was set so that 100% of the pork samples are identified as pork whereas in scenario 2, less than 100% of the pork samples were identified correctly improving the correct detection of non-pork samples.

To assist the halal certification process scientifically, scenario 1 is applicable, as the absence of pork is of prime importance. In this scenario using the NIR sensor (Table 3), non-pork samples are identified correctly at 27%, 51% and 53% in intact meat and 73% 42%, and 75% in ground meat samples for lamb, beef, and chicken, respectively. That means in some cases lamb, beef or chicken is wrongly identified as ‘containing pork’, which will lead to a rejection or further investigation of the sample. However, in no case will a consumer obtain a pork sample that was wrongly identified as being non-pork or in this case halal. When applying the Vis-NIR device, better results were achieved than with the NIR spectrometer (Table 3). Interestingly, only one pork sample was classified as ’out-of-class’ when applying the 2nd scenario. The same sample was also classified this way as being intact and ground. When using the NIR device, intact meat samples were more challenging to be detected as ground meat samples. This may be caused by the sample heterogeneity in intact meat and the more homogenous samples after the grinding procedure. Prieto et al. [10] reported similar observations and thus, recommended a high homogeneity for meat samples prior to spectroscopy measurements. The discrimination of species has been shown in the past by Mueller-Maatsch et al. [47], Schmurtzler et al. [48], Mamani-Linares et al. [49] and Restaino et al. [50] when using handheld Vis-NIR, FT-IR or benchtop NIR with a wavelength range above 2000 nm. Prieto et al. [10] suggested that the high wavelengths may be of great importance to discriminate species. Interestingly, the performance of the Vis-NIR sensor was better than the NIR sensor. This may be because of a different sampling procedure (i.e., pork samples deriving from the Netherlands) or because the wavelength range of the Vis-NIR covers better the relevant wavelengths.

### 3.4. Discriminant Models

When a sample in the OCC approach is detected as a halal meat species (out-of-class), the discriminant approach may be used for the discrimination of chicken, beef, lamb and pork samples.

#### 3.4.1. PLS-DA

**Figure 4:**
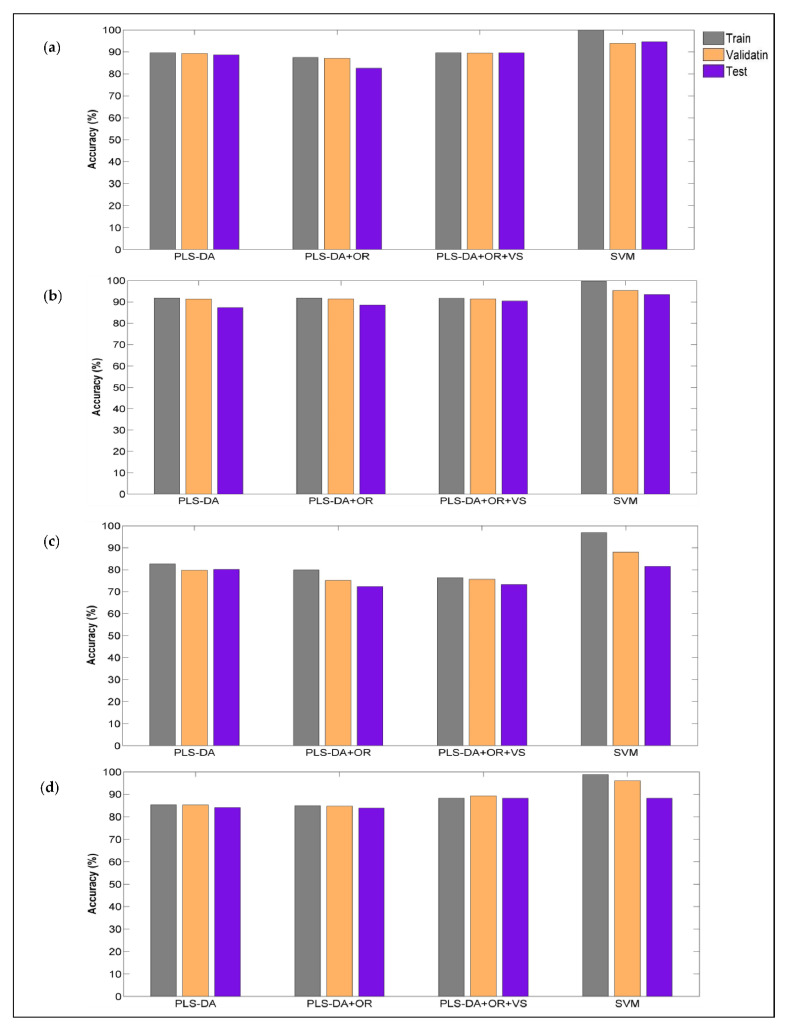
Classification accuracy of SVM and PLS-DA models in Vis-NIR and NIR sensors. Vis-NIR sensor: (**a**) intact meat samples; (**b**) ground meat samples. NIR sensor: (**c**) intact meat samples; (**d**) ground meat samples. Partial Least Squares Discriminant Analysis (PLS-DA); Outlier Removal (OR); Variable Selection (VS) and Support Vector Machine (SVM).

In a first step, the ability of PLS-DA models as a linear discrimination method to classify species was evaluated for the four classes (lamb, beef, chicken and pork). The most appropriate pre-processing strategy was chosen according to the highest sensitivity, specificity and lowest classification error (Tables S1 and S2). The best PLS-DA models for intact and ground meat measured with the Vis-NIR device were achieved with MSC (mean) plus gap segment 1st derivative (gap: 5, segment: 5) and EMSC, respectively. In this regard, the best total accuracy values for intact and ground meat were 94% and 92%, respectively (Figure 4a,b, Table S1). Furthermore, outlier detection using Q-residuals/Hotelling’s T^2^ and variable selection using a regression coefficient [37] was performed to improve PLS-DA classification. These methods slightly improved the model accuracy in ground meat (95%) but did not result in any improvement in intact meat (Figure 4a,b).

Also for the NIR sensor, the spectra were pre-processed with different methods and the best results (total accuracy) for intact meat spectra were achieved with median center plus gap segment 2nd derivative (gap: 5, segment: 5) and OSC (accuracy 85%) while MSC (mean) followed by 1st derivative (SavGol, filter width: 11 pt) provided the best results for ground meat spectra (accuracy 92%) (Figure 4c,d). Other attempts, such as outlier detection using Q-residuals/Hotelling’s T^2^ [33] and variable selection using variable importance in projection (VIP) with the “greater than one” rule [37] were performed to improve PLS-DA classification. These methods did not significantly improve the models’ performance (Figure 4c,d). Meat samples, especially those in the intact form have a heterogeneous nature that influences the performance of NIR spectroscopy prediction models. In previous studies, it has been reported that NIR spectroscopy prediction models were improved using ground versus intact (non-ground) meat samples [10,26,27]. Moreover, in the current study, it was shown that the grinding procedure reduced heterogeneity of meat samples and therefore improved the performance of the meat species classification model for both discriminant and OCC approaches in the NIR region (Table 3 and Table 4). Comparison between these two handheld sensors indicates when the Vis-NIR sensor is used, slightly better accuracy of the model can be achieved. This indicated that both pigment color information (from the visible region) and the composition of the muscle (from the NIR region) gave information to be used for identification purposes. These results are in agreement with other studies where the Vis-NIR spectra performed better, rather than only NIR spectra for speciation of different types of meats [43,51]. The results of different data splitting methods such as duplex, Kennard-stone and random algorithms were shown in Table S3 and the duplex method had relatively better performance [52]. Furthermore, the only difference between the three methods of cross-validation was related to the selection procedure of the samples, and similar results were obtained with all of the methodologies regarding latent variable selection and prediction ability (Table S4).

#### 3.4.2. SVM

SVM (with RBF and quadratic kernel function) is considered as a nonlinear machine learning method aiming to discriminate the species of different meat samples. The most appropriate pre-processing strategies for Vis-NIR were smoothing (SavGol, filter width: 15 pt, polynomial order: 0, derivative order: 0) plus SNV (weighted normalization) and Multiplicative Signal Correction (mean) for intact and ground meats, respectively. For the Vis-NIR sensor, an accuracy of 95% for intact meat and 93% for ground meat in the test set were reached (Figure 4a,b). For the NIR sensor, the best results (accuracy) for intact meat spectra were achieved with the MSC (mean) + 1st derivative (SavGol, order: 2, window: 15 pt) (accuracy 82%) while the gap segment 2nd derivative (gap: 5, segment: 5) provided the best results for ground meat spectra (accuracy 88%) (Figure 4a,d). The results showed SVM models with non-linear kernel function (RBF and quadratic) have relatively better performance rather than PLS-DA (as a linear model) for meat speciation (Table 4 and Table 5).

In the previous analyses, all four meat species samples were used for training the SVM model. Therefore, a final study was conducted based on a leave-class-out (LCO) methodology. Several SVM models were trained (and cross-validated) using 4 − 1 = 3 classes, while the left-out class was completely used as a test set. The classification accuracy of each four-class model was over 89% (Vis-NIR sensor) and 97% (NIR sensor) (Table 6 and Table 7). For both Vis-NIR and NIR sensors, the lamb spectra were classified mainly as either chicken (33% and 58%) or beef (59% and 33%), respectively. While other species (beef, chicken and pork) were allocated mainly as lamb using the Vis-NIR sensor; with the NIR sensor, the majority of chicken spectra (82%) were classified as lamb. Furthermore, the majority of beef spectra were allocated to lamb (54%) and pork (45%), whereas the greater number of pork spectra were assigned to lamb (38%) and beef (50%).

## 4. Conclusions

This is the first study regarding the development of methods for speciation of four meat species (intact and ground meat) using two handheld sensors combined with OCC and discriminant approaches. The presented OCC approach was, especially with the Vis-NIR sensor, suitable for the application of halal meat-species detection. The screening approach (OCC) with the handheld Vis-NIR and NIR can simplify increasing the number of samples to be evaluated and provide a fast-positive release while detecting the suspicious samples that are then forward for confirmatory methods. In discriminant analysis, Vis-NIR and NIR sensors were able to classify four meat species with promising classification accuracies (ranging from 81.5% to 94.7%) for intact and ground meat. The accuracy of the Vis-NIR sensor is higher than the NIR one and this is in agreement with the OCC approach.

These methods could be used as part of a two-tiered system by following up questionable results with a confirmatory method and therefore save both time and costs. The results are promising: for having more robust models, larger sample sets should be used to establish the actual potential of this technology with considering more breeds, cuts, age and sex. In summary, the presented approach is suitable for the application of halal meat-species detection in particular when the meat species is unknown as a screening test method. It should be useful for traders, shops, restaurants, or even consumers to ensure the safety and authenticity of their meat.

## Figures and Tables

**Table 1:** Details and AUROCs on the manually picked models for the NIR sensor.

	Model	Pre-Processing				Algorithm	AUROC			
		SNV	Derivative	Subset	DWT		Pork vs. lamb	Pork vs. beef	Pork vs. chicken	Pork vs. all
Intact	1	-	1st	3rd	-	SIMCA (3 PCs)	0.61	0.53	0.51	0.55
	2	DT	-	2nd	-	OCSVM	0.59	0.79	0.82	0.73
	3	SNV	-	3rd	-	PCA residual (3PCs)	0.54	0.75	0.82	0.70
Ground	1	-	1st	3rd	-	SIMCA (3 PCs)	0.93	0.73	0.84	0.83
	2	SNV	-	(full)	la8 (3–5)	OCSVM	0.80	0.85	0.78	0.81
	3	-	1st	4th	-	OCSVM	0.89	0.70	0.94	0.83

**Table 2:** Details and AUROCs on the manually picked models for the Vis-NIR sensor.

	Model	Pre-Processing				Algorithm	AUROC			
		SNV	Derivative	Subset	DWT		Pork vs. lamb	Pork vs. beef	Pork vs. chicken	Pork vs. all
Intact	1	-	1st	2nd	-	OCSVM	0.95	0.98	0.93	0.95
	2	DT	-	4th	-	SIMCA (3 PCs)	0.93	0.95	0.99	0.96
	3	SNV	-	(full)	la8 (3–5)	SIMCA (3 PCs)	0.93	0.93	0.98	0.95
Ground	1	-	1st	(full)	-	kNN (2 PCs)	0.97	0.98	0.98	0.98
	2	-	1st	4th	-	PCA residual (3 PCs)	0.96	0.98	0.96	0.97
	3	-	1st	(full)	-	SIMCA (3 PCs)	0.96	0.98	0.98	0.97

**Table 3:** Correct classification rate (%) of samples in two scenarios for the Vis-NIR and NIR sensors.

	VIS-NIR				NIR			
	Intact Meat		Ground Meat		Intact Meat		Ground Meat	
Scenario	1	2	1	2	1	2	1	2
Pork	100	98	100	98	100	79	100	79
Lamb	97	100	97	100	27	44	73	95
Beef	100	100	100	100	51	73	42	75
Chicken	100	100	95	100	53	85	75	88

**Table 4:** Classification performance (in %) of SVM (RBF kernel) model ^1^ for classification of lamb, beef, chicken and pork using six individual spectra of each sample for the NIR sensor.

		Intact Meat ^2^				Ground Meat ^3^		
		Train	CV	Test		Train	CV	Test
Lamb	Sensitivity	96.4	80.9	70.5		94.4	87.6	80.9
	Specificity	95.6	87.4	87.2		99.8	97.4	90.2
	Accuracy	96.0	84.0	78.4		97.0	92.3	85.4
	Error	4.0	16.0	21.6		3.0	7.7	14.6
								
Beef	Sensitivity	96.4	81.5	64.1		99.5	96.7	86.1
	Specificity	99.3	94.6	93.6		98.0	96.5	94.7
	Accuracy	97.8	87.8	77.4		98.7	96.6	90.3
	Error	2.2	12.2	22.6		1.3	3.3	9.7
								
Chicken	Sensitivity	98.2	89.2	84.7		100.0	95.1	81.2
	Specificity	99.5	96.7	91.9		99.8	99.0	94.6
	Accuracy	98.8	92.8	88.2		99.9	97.0	87.6
	Error	1.2	7.2	11.8		0.1	3.0	12.4
								
Pork	Sensitivity	91.2	78.5	74.0		99.3	97.4	83.3
	Specificity	99.6	98.0	91.2		100.0	99.2	96.8
	Accuracy	95.3	87.7	82.1		99.6	98.3	89.8
	Error	4.7	12.3	17.9		0.4	1.7	10.2

^1^ Cross validation (CV): Venetian blinds (number of data split: 10, thickness: 1). ^2^ Pre-processing: MSC (mean) + 1st derivative (SavGol) (order: 2, window: 15 pt). ^3^ Pre-processing: gap segment 2nd derivative (gap: 5, segment: 5).

**Table 5:** Classification performance (in %) of SVM (kernel function: quadratic) model ^1^ for classification of lamb, beef, chicken and pork using six individual spectra of each sample for the Vis-NIR sensor.

		Intact Meat ^2^				Ground Meat ^3^		
		Train	CV	Test		Train	CV	Test
Lamb	Sensitivity	100.0	79.4	80.7		99.2	92.7	92.8
	Specificity	100.0	96.0	96.0		100.0	93.6	88.3
	Accuracy	100.0	87.3	88.0		99.6	93.2	90.5
	Error	0.0	12.7	12.0		0.4	6.8	9.5
								
Beef	Sensitivity	100.0	85.4	93.7		100.0	85.6	83.3
	Specificity	100.0	93.1	93.5		99.7	98.1	97.6
	Accuracy	100.0	89.1	93.6		99.8	91.6	90.2
	Error	0.0	10.8	6.4		0.2	8.4	9.8
								
Chicken	Sensitivity	100.0	98.9	94.7		100.0	94.4	87.5
	Specificity	100.0	100.0	100.0		100.0	99.8	99.4
	Accuracy	100.0	99.4	97.3		100.0	97.0	93.2
	Err	0.0	0.6	2.7		0.0	3.0	6.8
								
Pork	Sensitivity	100.0	100.0	100.0		100.0	100.0	100.0
	Specificity	100.0	100.0	100.0		100.0	100.0	100.0
	Accuracy	100.0	100.0	100.0		100.0	100.0	100.0
	Error	0.0	0.0	0.0		0.0	0.0	0.0

^1^ Cross validation (CV): 5-fold cross validation. ^2^ Pre-processing: smoothing (SavGol) (filter width: 15 pt) + Standard Normal Variate (SNV). ^3^ Pre-processing: Multiplicative Scatter Correction (mean).

**Table 6:** SVM classification performance in leave-class-out validation. Accuracy values (column 2) are based on five-fold cross-validation of the remaining three class data (ground meat samples) for the Vis-NIR sensor.

Left Out	Accuracy (%)	Spectra	Lamb	Beef	Chicken	Pork
Lamb	99.8	222	--------------	131 (59 %)	75 (33.8 %)	16 (7.2 %)
Beef	99.0	240	231 (96.3 %)	------------	7 (2.9 %)	2 (0.8 %)
Chicken	92.1	240	220 (91.7 %)	2 (0.8 %)	-----------	18 (7.5 %)
Pork	89.0	240	227 (94.6 %)	0 (0.0 %)	13 (5.4 %)	------------

**Table 7:** SVM classification performance in leave-class-out validation. Accuracy values (column 2) are based on Venetian blinds (number of data split: 10, thickness: 1) cross-validation of the remaining three class data (ground meat samples) for the NIR sensor.

Left Out	Accuracy (%)	Spectra	Lamb	Beef	Chicken	Pork
Lamb	99.3	246	------------	82 (33.3%)	145 (58.9%)	19 (7.7%)
Beef	97.9	288	155 (53.8%)	--------------	4 (1.3%)	129 (44.8%)
Chicken	97.4	240	197 (82.0 %)	10 (4.1%)	------------	33 (13.7%)
Pork	97.4	204	77 (37.7 %)	101 (49.5 %)	26 (12.7 %)	------------

## Data Availability

Not applicable.

## Supplementary Materials

The following supporting information can be downloaded at: https://www.mdpi.com/article/10.3390/foods11010071/s1, Table S1: Classification performance (in %) of PLS-DA model1 for classification of Lamb, Beef, Chicken and Pork using 6 individual spectrum of each sample for the Vis-NIR sensor, Table S2: Classification performance (in %) of PLS-DA model1 for classification of Lamb, Beef, Chicken and Pork using 6 individual spectrum of each sample for the NIR sensor, Table S3: Effect of different data splitting on classification performance (%) of PLS-DA model1 in ground meat samples, Table S4: Effect of different cross-validation on classification performance (%) of PLS-DA model1 in ground meat samples.
