# Supplementary material for: The Feasibility of Two Handheld Spectrometers for Meat Speciation Combined with Chemometric Methods and Its Application for Halal Certification
Source: Foods. 2021 Dec 29;11(1):71. doi: 10.3390/foods11010071 (PMC8750306; doi:10.3390/foods11010071)
Supplement: Supplementary file 1 [file foods-11-00071-s001.zip › foods-1495223-supplementary.pdf]

Supplementary Materials

Table S1: Classification performance (in %) of PLS-DA model[1] for classification of Lamb, Beef, Chicken and Pork using 6 individual spectrum of each sample for the Vis-NIR sensor.

		Intact meat[2]			Ground meat[3]		
		Train	CV	Test	Train	CV	Test
Lamb	Sensitivity	98.6	98.6	100.0	97.1	95.7	100.0
	Specificity	61.0	60.7	71.1	65.5	65.3	58.1
	Accuracy	79.9	79.7	85.6	81.3	80.5	79.1
	Error	20.1	20.3	14.4	18.7	19.5	20.9
Beef	Sensitivity	92.2	92.2	87.5	95.4	95.4	98.5
	Specificity	81.4	81.0	77.8	86.6	86.4	71.3
	Accuracy	86.8	86.6	82.7	91.0	91.0	84.9
	Error	13.2	13.4	17.3	9.0	9.0	15.1
Chicken	Sensitivity	96.5	95.1	91.7	97.9	97.9	97.9
	Specificity	94.4	94.6	92.5	98.3	98.3	97.8
	Accuracy	95.5	94.9	92.1	98.1	98.1	97.9
	Error	4.5	5.1	7.9	1.9	1.9	2.1
Pork	Sensitivity	96.7	96.7	91.7	96.6	96.6	77.8
	Specificity	95.8	95.6	96.8	96.9	96.9	98.0
	Accuracy	96.3	96.2	94.3	96.8	96.8	87.9
	Error	3.7	3.8	5.7	3.2	3.2	12.1

[1] Cross-validation (CV): Venetian blinds (Number of data split: 10, thickness: 1)

[2] PLS-DA (LVs: 4) Preprocessed with MSC (mean) + Gap segment 1st derivative

[3] PLS-DA (LVs: 4) Preprocessed with Extended Multiplicative Scatter/Signal Correction (EMSC)

Table S2: Classification performance (in %) of PLS-DA model[1] for classification of Lamb, Beef, Chicken and Pork using 6 individual spectrum of each sample for the NIR sensor.

		Intact meat[2]			Ground meat[3]		
		Train	CV	Test	Train	CV	Test
Lamb	Sensitivity	76.8	66.1	73.1	83.3	82.7	98.8
	Specificity	78.1	74.6	79.9	68.4	68.0	60.6
	Accuracy	77.5	70.4	76.5	76.0	75.4	80.0
	Error	22.5	29.6	23.5	24.0	24.6	20.0
Beef	Sensitivity	85.1	82.1	80.8	89.4	88.0	91.7
	Specificity	83.7	82.5	75.5	83.8	83.5	93.9
	Accuracy	84.5	82.3	78.2	86.6	85.8	92.8
	Error	15.5	17.7	21.8	13.4	14.2	7.2
Chicken	Sensitivity	89.3	87.5	81.9	99.3	98.6	94.8
	Specificity	90.1	88.5	88.1	89.0	89.3	77.5
	Accuracy	89.7	88.0	85.1	94.2	94.0	86.2
	Error	10.3	12.0	14.9	5.8	6.0	13.8
Pork	Sensitivity	77.2	78.5	79.6	85.3	85.9	97.9
	Specificity	81.3	78.6	80.7	84.7	84.7	89.7
	Accuracy	79.3	78.6	80.2	85.0	85.3	93.8
	Error	20.7	21.4	19.8	15.0	14.7	6.2

[1] Cross validation: Venetian blinds (Number of data split: 10, thickness: 1)

[2] PLS-DA (LVs: 9) Preprocessed with Median center + Gap segment 2nd derivative + OSC (Orthogonal Signal Correction)

[3] PLS-DA (LVs: 5) Preprocessed with MSC (mean) + 1st derivative (SavGol) (order: 2, window: 11 pt)

Table S3: Effect of different data splitting on classification performance (%) of PLS-DA model[1] in ground meat samples:

		Duplex algorithm			Kennard-stone algorithm			Random algorithm (average of 3 repeats)		
		Train	CV[2]	Test	Train	CV	Test	Train	CV	Test
Lamb	Sensitivity	83.3	82.7	98.8	88.5	88.0	64.8	87.0	86.0	83.4
	Specificity	68.4	68.0	60.6	71.7	71.5	76.9	70.5	70.6	69.0
	Accuracy	76.0	75.4	80.0	80.2	79.8	70.9	78.9	78.4	76.3
	Error	24.0	24.6	20.0	19.8	20.2	29.1	21.1	21.6	23.7
Beef	Sensitivity	89.4	88.0	91.7	87.2	87.2	92.6	91.2	91.0	81.2
	Specificity	83.8	83.5	93.9	85.5	84.9	81.7	87.7	87.5	86.6
	Accuracy	86.6	85.8	92.8	86.4	86.1	87.2	89.5	89.4	84.0
	Error	13.4	14.2	7.2	13.6	13.9	12.8	10.5	10.6	16.0
Chicken	Sensitivity	99.3	98.6	94.8	96.1	95.6	98.3	95.0	95.2	93.8
	Specificity	89.0	89.3	77.5	88.8	88.2	83.8	86.9	87.0	86.3
	Accuracy	94.2	94.0	86.2	92.5	91.9	91.1	91.1	91.3	90.2
	Error	5.8	6.0	13.8	7.5	8.1	8.9	8.9	8.7	9.8
Pork	Sensitivity	85.3	85.9	97.9	92.0	92.0	80.3	86.8	86.4	88.2
	Specificity	84.7	84.7	89.7	86.4	86.2	84.7	85.2	85.0	88.2
	Accuracy	85.0	85.3	93.8	89.3	89.2	82.5	86.1	85.9	88.3
	Error	15.0	14.7	6.2	10.7	10.8	17.5	13.9	14.1	11.7

[1] Pre-processing: MSC (mean) + 1st derivative (SavGol) (order: 2, window: 11 pt)

[2] CV: cross validation

Table S4: Effect of different cross-validation on classification performance (%) of PLS-DA model[1] in ground meat samples:

		Venetian blinds[2]			Leave one out			Random subsets[3]		
		Train	CV	Test	Train	CV	Test	Train	CV	Test
Lamb	Sensitivity	83.3	82.7	98.8	83.3	83.3	98.8	83.3	83.1	98.8
	Specificity	68.4	68.0	60.6	68.4	68.4	60.6	68.4	68.0	60.2
	Accuracy	76.0	75.4	80.0	75.9	75.9	79.8	75.9	75.6	79.8
	Error	24.0	24.6	20.0	24.1	24.1	20.2	24.1	24.4	20.2
Beef	Sensitivity	89.4	88.0	91.7	89.9	87.5	91.7	89.9	87.4	91.7
	Specificity	83.8	83.5	93.9	83.8	83.8	93.9	83.8	83.6	93.9
	Accuracy	86.6	85.8	92.8	86.6	85.7	92.8	86.6	85.6	92.8
	Error	13.4	14.2	7.2	13.4	14.3	7.2	13.4	14.4	7.2
Chicken	Sensitivity	99.3	98.6	94.8	99.3	99.3	94.8	99.3	99.1	94.8
	Specificity	89.0	89.3	77.5	89.0	89.0	77.5	89.0	88.9	77.5
	Accuracy	94.2	94.0	86.2	94.2	94.2	86.2	94.2	94.0	86.2
	Error	5.8	6.0	13.8	5.8	5.8	13.8	5.8	6.0	13.8
Pork	Sensitivity	85.3	85.9	97.9	85.3	84.6	97.9	85.3	83.9	97.9
	Specificity	84.7	84.7	89.7	84.9	84.5	89.7	84.7	84.6	89.7
	Accuracy	85.0	85.3	93.8	85.0	84.6	93.8	85.0	84.3	93.8
	Error	15.0	14.7	6.2	15.0	15.4	6.2	15.0	15.7	6.2

[1] Pre-processing: MSC (mean) + 1st derivative (SavGol) (order: 2, window: 11 pt)

[2] Number of data split: 10, thickness: 1

[3] Number of data split: 10, number of iterations: 20
